# Establishment of a Real-Time Quantitative PCR Assay for Porcine Circovirus-Like Virus and the First Evidence of Its Spread to Hainan and Jiangxi Provinces of China

**DOI:** 10.3389/fvets.2022.853761

**Published:** 2022-06-21

**Authors:** Leyi Zhang, Xinming Zhang, Ge Xu, Lin Wang, Xianhui Liu, Pengfei Zhang, Shuangyun Wang, Tairun Liang, Zhipeng Wang, Yanling Liu, Zheng Xu, Zan Li, Guojun Huang, Changxu Song

**Affiliations:** ^1^National Pig Breeding Industry Engineering and Technical Research Center, College of Animal Science and National Engineering Center for Swine Breeding Industry, South China Agriculture University, Guangzhou, China; ^2^Lingnan Modern Agricultural Science and Technology Guangdong Laboratory, Guangzhou, China; ^3^Dongrui Food Group Co. Ltd, Heyuan, China; ^4^Daguang Food Group Co. Ltd, Jiangmen, China

**Keywords:** porcine circovirus-like virus, real-time quantitative PCR assay, Jiangxi and Hainan Province, epidemic, mutation

## Abstract

Porcine Circovirus-like (PCL) virus, a new emerging virus, has been widely detected in Guangdong, Guangxi, and Anhui provinces in China, which may be a novel agent causing severe diarrhea in newborn piglets and tending to spread widely. Evidence suggests that the virus is related to hemorrhagic enteritis and diarrhea, and many newborn piglets were emaciated to death after infection. Therefore, a sensitive, quick, and accurate detection system for virus detection and epidemiological investigation is necessary. In this study, we developed a real-time quantitative PCR assay based on SYBR green for the detection of PCL virus. The ORF4 conserved region of PCL virus was found by the alignment of the uploaded genome sequences to design specific primers, and the primers were tested and showed good specificity, sensitivity, and reproducibility. Approximately, 138 fecal samples were obtained from diarrheal pigs in South China from June to December 2021. Approximately, 22.46% (31/138) of the samples and 40% (8/20) of the pig farms were positive for PCL virus, respectively, by using this method. Moreover, it is worth noting that the virus was first detected in Hainan and Jiangxi Provinces of China, which means that the virus may spread widely in China. Through evolutionary tree analysis and partial sequence comparison, there are some differences of virus genes in each province, suggesting that there is a risk of variation, and the four PCL virus strains showed a sequence similarity of 86.7%–87.8% for the rep gene and 92.2%–92.9% for the Rep protein, respectively, with Bo-Circo-like virus that is detected in bovine, which further demonstrates a close relationship between the two viruses that originated from different animals. In conclusion, our study provides a useful diagnostic approach to PCL virus detection and epidemiological inquiry. Meanwhile, the epidemic data using this real-time qPCR assay provide evidence for the widespread variations and epidemic of the virus in South China, and warn the appropriate measures for prevention, and control of porcine circovirus-like virus infection should be under consideration in pig production.

## Introduction

Viruses with tiny circular Rep-encoding ssDNA (CRESS DNA) genomes, which typically contain a replication associated protein (Rep), are extensively found in nature (1, 2), and have been found and reported in humans (3, 4), cattle (5), pigs (6–8), dogs (9), ducks (10), and other animals with varying degrees of clinical symptoms and damage. At present, among these viruses that infected pigs and caused serious economic loss, Porcine Circovirus type 2 (PCV2), Porcine Circovirus type 3 (PCV3), and recently discovered virus Porcine Circovirus type 4 (PCV4) have brought serious harm to the pig industry (11–13). PCV2 is mainly related to porcine dermatitis, and nephropathy syndrome (PDNS) (14), PCV2, and PCV3 are the main pathogen causing postweaning multisystemic wasting syndrome (PMWS) (12). The pathogenicity of PCV4 remains to be further verified (15). The above viruses seriously affect the health of pigs and have been a hindrance to the development of the pig industry. In the face of the problems caused by these viruses, at the same time, newCRESS viruses also have been emerging in an endless stream. Recently, a new circovirus Porcine Circovirus-like (PCL) virus has closely associated with piglet hemorrhagic enteritis, and diarrhea is gaining further prevalent in south China, which is a single-stranded DNA virus with a genome of 3,944 nucleotides encoding the replication protein and the non-classic capsid protein (8). The virus has been reported in Guangxi (16), Guangdong (8), and Anhui (17) provinces, causing varying degrees of clinical symptoms.

In 1998, in the lung samples of pigs with post-weaning multisystem wasting syndrome collected from a farm in the United States, viruses were isolated and cultured in PK-15 cells. Electron microscopy showed that the new virus particles of 17 nm in diameter is similar to PCV in morphology, and named as Porcine Circovirus-like virus (18). Until 2020 in Guangxi, China, three more PCL viruses were found in intestinal tissue samples of diarrheal pigs (16). Subsequently, we detected this virus in samples from three diarrhea pig farms in Guangdong province (8). Followed by decades of evolution, the virus may become more prevalent and its symptoms turn into more pronounced. Therefore, we conducted an epidemiological investigation for pig herds in south China. Upon further investigation, sufficient evidence demonstrated that the virus has undeniable association with piglet severe diarrhea and hemorrhagic enteritis and leads to wasting and death of piglets. However, sows carrying the virus show no symptoms. According to the autopsy results, the virus may cause submaxillary, Inguinal and intestinal lymph nodes swelling, or with high temperature, mesenteric hemorrhage, granulomatous interstitial pneumonia (17). However, in terms of spreading pressure on the virus, no specific PCL virus detection assay has yet been developed. Therefore, in order to better target the virus for surveillance and epidemiological investigation and to further limit the economic loss that may be caused by the virus, it is critical to develop an effective, clinical, and sensitive detection approach.

In this study, we established a SYBR green-based real-time qPCR detection method for PCL virus, providing a methodological basis of the detection and epidemiological investigation for the virus, which will be also useful for the further understanding of the virus genetic characteristics.

## Materials and Methods

### Viruses and Clinical Samples

Porcine Epidemic Diarrhea Virus (PEDV), Porcine Parvovirus (PPV), Porcine Pseudorabies Virus (PRV), Transmissible Gastroenteritis Virus (TGEV), Porcine Reproductive and Respiratory Syndrome Virus (PRRSV), PCV2 strains were isolated and preserved by the National Pig Breeding Swine Disease Control Laboratory of the National Pig Breeding Industry Engineering Technology Research Center; the positive nucleic acid of PCV3 was derived from the lung samples of pigs in Kaiping, Guangdong. From June to August 2021, clinical samples (stool, gut, and other materials) were collected from 138 cases of diarrhea pigs from 20 different farms in Guangdong, Jiangxi, and Hainan provinces. The detailed information of clinical samples is shown in **Table 3**.

### Extraction of Viral Nucleic Acids and Reverse Transcription

The samples were homogenized with sterilized PBS. The supernatant was taken after centrifugation; the RaPure Viral RNA/DNA Kit (Magen, Shanghai, China) was used to extract viral nucleic acid. Using the PrimeScript™ reagent Kit with gDNA Eraser (Takera, China), the viral RNA (TGEV, PRRSV, and PEDV) was reverse transcribed for cDNA. The experimental procedures are strictly conducted in accordance with the product instructions.

### Primer Design

The nucleotide sequences of PEDV (GenBank: MK458329.1), PPV (GenBank: AF257079), TGEV (GenBank: U26210.1), PRV (GenBank: AF257079), PRRSV (GenBank: EU926974.1), PCV2 (GenBank: HM038024.1), and PCV3 (GenBank: MH491016.1) were downloaded from GenBank. Primers are designed on the Primer3website and Synthesized by Sangon company (Shanghai, China). Porcine circovirus-like virus (PCLV) ORF4 sequences with fewer mutations were selected for primer design by comparing published PCLV genes (**Figure 7**). The Basic Local Alignment search tool was used to validate all primers. Primer sequence as shown in Table 1.

**Table 1:** Primers sequences information.

**Name**	**Assay**	**Sequence**	**Length (bp)**
PCLV-ORF4-F1 (*Eco*RI)	PCLV ORF4 gene plasmid construction	TGGAGGCCCGAATTCGGTCG ATGTTCAGTGAAATCACTCC	637
PCLV-ORF4-R1 (*Kpn*I)		GTACCTCGAGAGATCTCGGT TTATTCAAAGAGCGCGGAAA	
PCLV-RT-F1	Real-time PCR	CTGCAAAGGAGACGTCATGG	229
PCLV-RT-R1		GTACTTGATCCAAGCGGAAA	
PCLV-F1	cPCR	TAGACTATCATGGCGCTGGG	181
PCLV-R1		TCATTTCCTGCGGCTTGAAC	
PCLV-F2	cPCR	CGCGCAATACGTTGGTGTTT	534
PCLV-R2		CGACTTGCCTTTACCAGGTG	
PCV2-F	cPCR	GGTCGTATATACTGTTTTCG	583
PCV2-R		GGGGCGTCGGTAGAACCGGT	
PCV3-F	cPCR	TACTACACAAAGAAATACTC	491
PCV3-R		ACTCTTCAGACAGTAAGGTC	
PPV-F	cPCR	GGCACGTTGATCCTCCGTCA	381
PPV-R		ATTTTTCTTAGAAGCCGTCT	
TGEV-F	cPCR	TTGCATGGAGCTAGTTACCG	600
TGEV-R		GAACTCTCGACACGGCAACA	
PRV-F	cPCR	ACGAGCCCCGCTTCCACGCG	700
PRV-R		CACCGGTCGCCGAGCAGCGG	
PEDV-F	cPCR	ATGGCTTCTGTCAGTTTTCA	660
PEDV-R		CAGTCCCAAAAGCGGTTATG	
PRRSV-F	cPCR	GCAACAAATCTTGAAGAATG	930
PRRSV-R		CCCTCTCCGGAGTATAAGTC	

### Standard Plasmid Construction

As a template, nucleic acids isolated from PCLV positive clinical samples were employed. PCLV-ORF4-F1 (*Eco*RI) and PCLV-ORF4-R1 (*Kpn*I) primers with restriction sites were used for PCR amplification according to the extracted template, and the length of PCLV ORF4 with 597 bps was obtained and cloned into PCMV-HA (N) vector.

### Assay for Quantitative PCR

The qPCR detection system for PCLV was 2× Taq PCR Master Mix II (Tiangen, China) 12.5 μl, ddH_2_O 8.5 μl, PCLV-F primer 1 μl (10 mM), PCLV-R primer 1 μl (10 mM), and template 2 μl. PCR was performed under the following conditions: after treatment at 95°C for 5 min, 35 cycles of 95°C for 15 s, 56°C for 30 s, 72°C for 60 s, then 72°C for 10 min, 12°C for 5 min, 1% agarose gel electrophoresis was used to validate PCR products.

### The Development of the Real-Time PCR Assay

Eastep® qPCR Master Mix (2×; promega, USA) was used to perform the real-time PCR assay and conducted in the Applied BiosystemsStepOnePlus Real-Time PCR System (Life Technologies). The assay system includes 10 μl of Eastep® qPCR Master Mix (2×), 2 μl of the template, 4 μl of 10-mM PCLV-F2 primer and PCLV-R2 primer, 7 μl of nuclease-free water, 2 μl of CXR 100 x^*^, 95°C for 120 s, 40 cycles of 95°C for 15 s; finally, 60°C for 40–60 s. In the range of 65–95°C. The melting curve was analyzed by collecting a SYBR green fluorescent signal.

### Standard Curve Formulation

The standard curves for qPCR were established using 1.7 × 10^8^-1.7 × 10^2^ copies/μl PCMV-HA(N)-PCLV-ORF4 at different concentrations. Each group of samples was repeated three times, and the corresponding Ct value was measured by collecting the fluorescence signal, and then, consequently, the amplification curve was obtained. The standard curve was drawn with the Ct value of each plasmid concentration as the ordinate and the logarithm of standard plasmid copy number as the abscissa.

### Analytical Sensitivity Test

The sensitivity of this assay was compared with that of the conventional PCR method. The constructed PCMV-HA (N)-PCLV-ORF4 plasmid and the positive sample nucleic acid were diluted 10 times for detection. The detection limit of real-time quantitative PCR was defined as the minimum plasmid concentration and minimum nucleic acid dilution concentration of the positive sample that can be quantified and amplified by this method and maintained within the linear range of the standard curve. The limit of detection (LoD) of conventional PCR was the lowest plasmid concentration observed in the band detected by agarose gel electrophoresis. The lowest detection concentration of the two methods was compared.

### Specificity Analysis

In order to explore the specificity of this method, the ddH_2_O was used as the negative control, the DNA of PPV, PCV2, PCV3, and PRV, and the cDNA of TGEV, PRRSV, and PEDV were used as templates for amplification.

### Reproducibility Analysis

Using plasmid PCMV-HA(N)-PCLV-ORF4 as a template, the reproducibility of SYBR Green real-time fluorescence quantitative PCR was evaluated with three concentration gradients (1.7 × 10^8^ copies/μl, 1.7 × 10^6^ copies/μl, and 1.7 × 10^4^ copies/μl). The assay was performed in three periods with five repeats per sample. By analyzing the variation of Ct values in different replicates, the coefficient of variation (CV) was calculated by dividing the standard deviation (SD) by the mean.

### Detection of PCLV in Clinical Specimens

The accuracy of PCLV detection in clinical samples was based on this method. We used the RaPure virus RNA/DNA kit (Magen, China) to extract viral nucleic acid for detection. A Ct value <35 is considered positive for PCLV. The conventional PCR was used for further validation, and the results were compared. All positive specimens were sequenced.

### Phylogenetic Analysis

The ORF1 sequences of the Chinese and American endemic strains were downloaded from GenBank. Bo-Circo-like virus, identified from a calf with severe hemorrhagic enteritis in China, its ORF1 sequences, was also downloaded from GenBank, and the serial numbers were displayed in the evolutionary tree. All permutations were further aligned with MegAlign (Lasergene) using ClustalW alignment. MEGA7 software was used to construct the phylogenetic tree with 1,000 bootstrap repeats using the maximum likelihood method.

## Results

### Amplification Plot, Curve Analysis, and Standard Curve Melting

**Figure 1:**
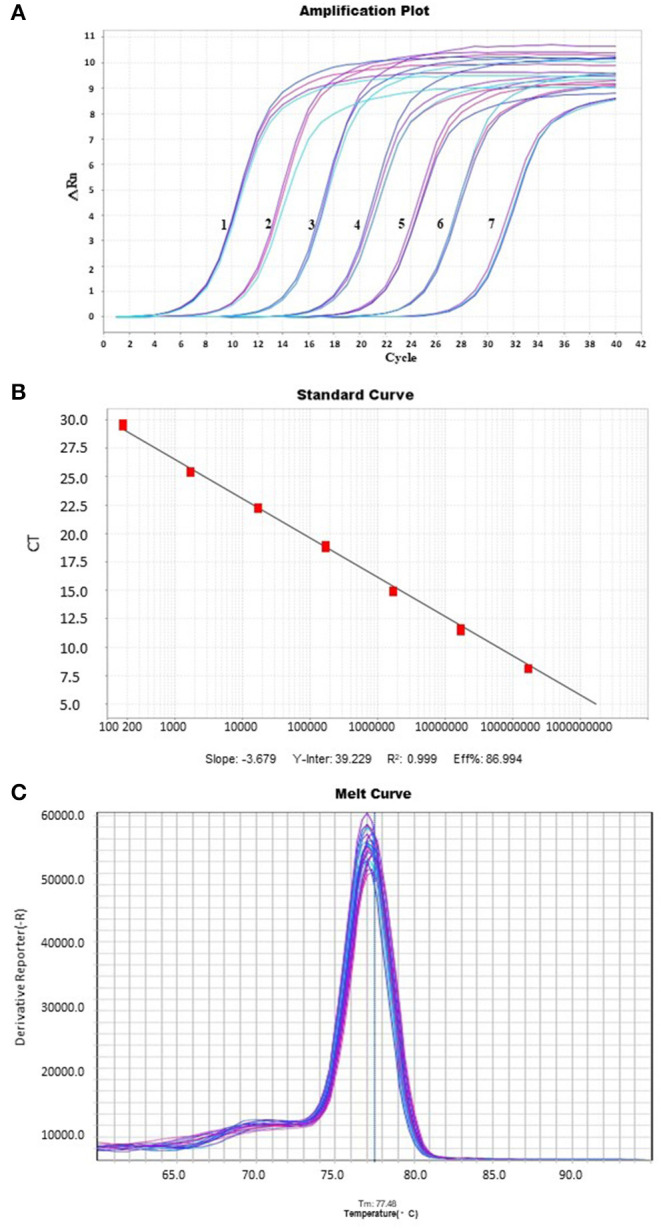
An amplification plot, melting curve analysis, and a standard curve. Real-timeq PCR detection of PCL virus. **(A)** A plot of amplification: The cycle threshold (Ct) values, the *Y*-axis represents the fluorescence intensity, and 1–8 show plasmid concentrations, ranging from 1.7 × 10^2^ copies/μl to 1.7 × 10^8^ copies/μl. **(B)** Standard Curve: The *X*-axis represents the copy number, which ranges from 1.7 × 10^2^ copies/μl to 1.7 × 10^8^ copies/μl. The corresponding Ct values are represented on the *Y*-axis. **(C)** The melting curve with a melting peak at 77.48 ± 0.5°C. The peak is single and of good quality.

The amplification curve showed good repeatability, and the fluorescence intensity changed regularly with the multiple dilution of plasmid concentration (Figure 1A). The coefficient of determination was 0.999, while the slope of the standard curve was −3.679, the *Y*-intercept was 39.229, and the amplification efficiency was estimated to be 86.994%. The linear relationship is well presented (Figure 1B). According to the dissolution curve, all positive samples were unimodal, and the melting temperature was 77.48 ±.5°C (Figure 1C). At the same time, no melting curve or dimer curve was collected in the negative control.

### Analytical Sensitivity Test

**Figure 2:**
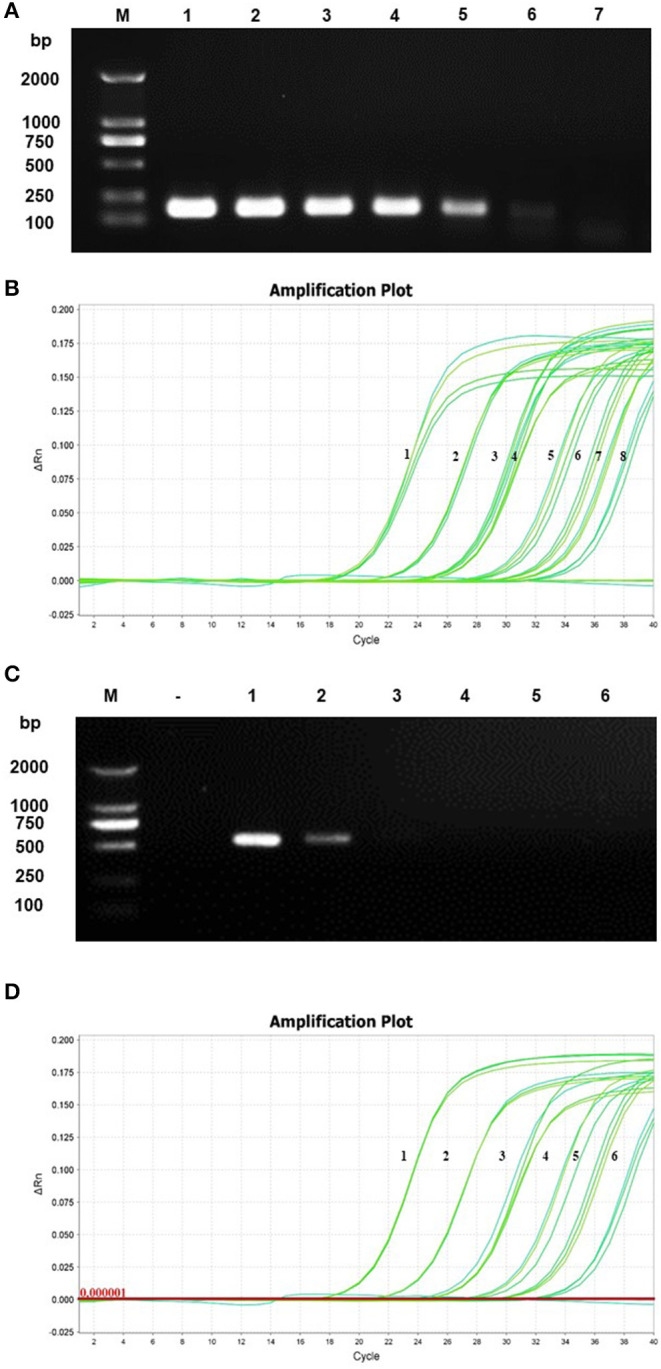
Sensitivity analysis. The constructed PCMV-HA (N)-PCLV-ORF4 plasmid and the positive sample nucleic acid were diluted 10 times for detection. The conventional PCR and real-time qPCR were used to detect the diluted plasmid **(A,B)** and positive nucleic acid **(C,D)**, respectively; serial numbers represent different dilutions.

To detect the dilutions of PCMV-HA (N)-PCLV-ORF4 (1.7 × 10^8^ copies/μl−1.7 × 10^2^ copies/μl) and the positive sample nucleic acid by using conventional PCR showed that the minimum plasmid concentration was 1.7 × 10^3^ copies/μl, and the minimum nucleic acid dilution concentration of positive samples was 10^−3^ (Figures 2A,C). Based on the analysis of the amplification curve and the standard curve, the LoD detected by real-time qPCR was 1.7 × 10^1^ copies/μl plasmid concentration of 10^−6^ (Figures 2B,D). Therefore, real-time qPCR was 100 to 1,000 times more sensitive than that of conventional PCR.

### Specificity Analysis

**Figure 3:**
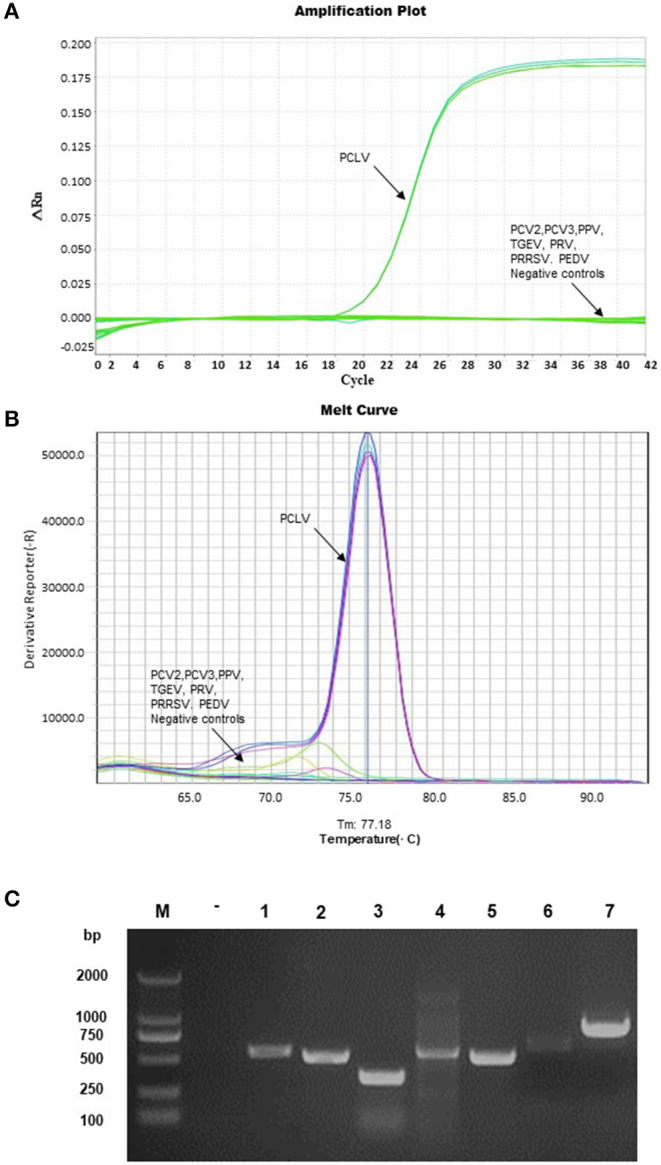
Specificity analysis. The DNA of PPV, PCV2, PCV3, and PRV; the cDNA of TGEV, PRRSV, and PEDV were used as templates for amplification. The amplification plot and melting curve analysis as shown in the figures **(A,B)**. The nucleic acid as a template was detected by the conventional PCR, and the nucleic acid of each virus was successfully extracted; the sequence of samples represented negative control, PCV2, PCV3, PPV, PRV, TGEV, PEDV, and PRRSV **(C)**.

Only the PCLV group collected a strong fluorescence signal (Figure 3A) and a specific melting peak as shown at 77.18 ± 0.5°C through specificity analysis (Figure 3B). No signal amplification was found in the nucleic acid of PCV2, PCV3, PPV, TGEV, PRV, PRRSV, and PEDV, which were extracted successfully (Figure 3C). Both Ct values were >35, and there was no distinct melting peak (Figures 3A,B). The method showed high specificity in clinical sample detection and repeatability detection.

### Reproducibility Analysis

Reproducibility analysis of this method and the intra-assay SD and CV were 0.04–0.14% and 0.37–0.78%, respectively. The SD and CV between assays were 0.053–0.143% and 0.042–0.139% (Table 2). These results indicated that the real-time PCR assay is highly reproducible.

**Table 2:** Intra- and inter-assay reproducibility for real-time PCR.

**Concentration of standard plasmid (copies/μl)**	**Intra-assay variability**			**Inter-assay variability**		
	**Mena (Cq)**	**SD**	**CV (%)**	**Mena (Cq)**	**SD**	**CV (%)**
1.7 × 10^8^	6.261	0.063	1.00	6.294	0.139	2.20
1.7 × 10^6^	13.972	0.053	0.37	13.940	0.081	0.58
1.7 × 10^4^	20.701	0.143	0.69	20.729	0.042	0.20

### Clinical Sample Detection and Geographic Distribution of Pigs With Porcine Circovirus-Like Virus in China

**Figure 4:**
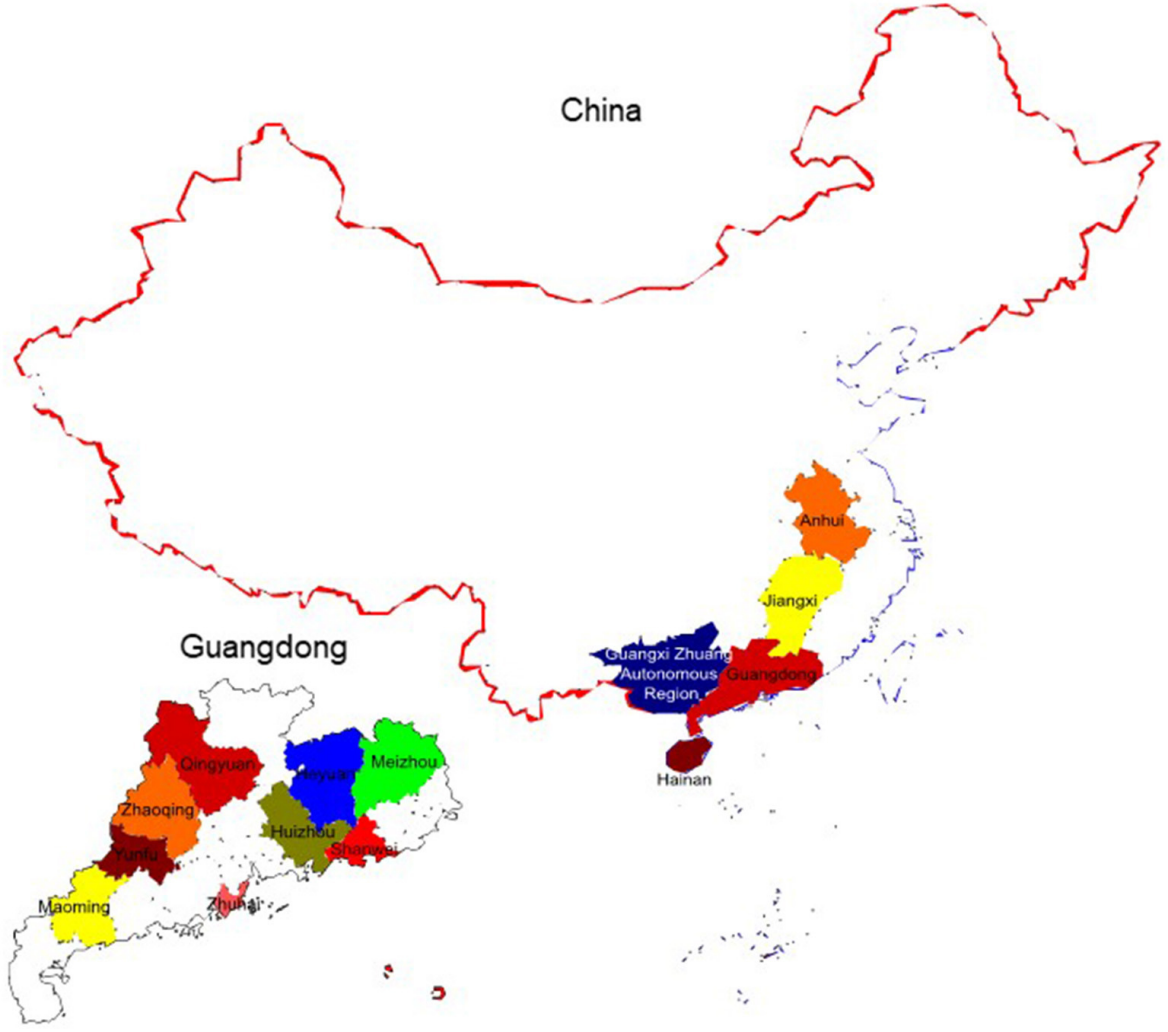
The geographic distribution maps of Porcine Circovirus-like Virus in all provinces of China and cities of Guangdong province were made based on the reported results and our findings. The colors indicate the locations of farms where Porcine Circovirus-like virus has been identified.

According to the results of clinical samples from six positive pig farms, the positive detection rate using real-time qPCR and conventional PCR was 22.46% (31/138) and 17.39% (24/138), respectively. Sequencing was used to confirm all positive samples (Table 3). The geographic distribution maps of porcine circovirus-like viruses in all provinces of China and cities of Guangdong province were made based on the reported results and our findings. The color-coded area indicates the locations of farms where porcine circovirus-like virus has been identified (Figure 4). The results showed that positive samples were detected in China's Hainan and Jiangxi provinces, as well as Yunfu, Huizhou, and Shanwei cities in Guangdong Province, indicating that the virus has a tendency to spread.

**Table 3:** Statistics of PCLV positive materials from June to August 2021.

**Sampling time**	**Sampling position**	**Number of samples**	**Sample types**	**Number of positive sample**	
				**Real-time RCR**	**cPCR**
6.28	Hainan Province	38	Feces	10	8
6.30	Yunfu City	27	Stillbirth, cord blood	2	1
7.1	Heyuan City	18	Feces	5	3
7.16	Shanwei City	13	Feces	4	4
7.20	Jieyang City	17	Feces	8	8
8.9	Jiangxi Province	25	Feces, small intestine	2	0

### Phylogenetic Analysis

**Figure 5:**
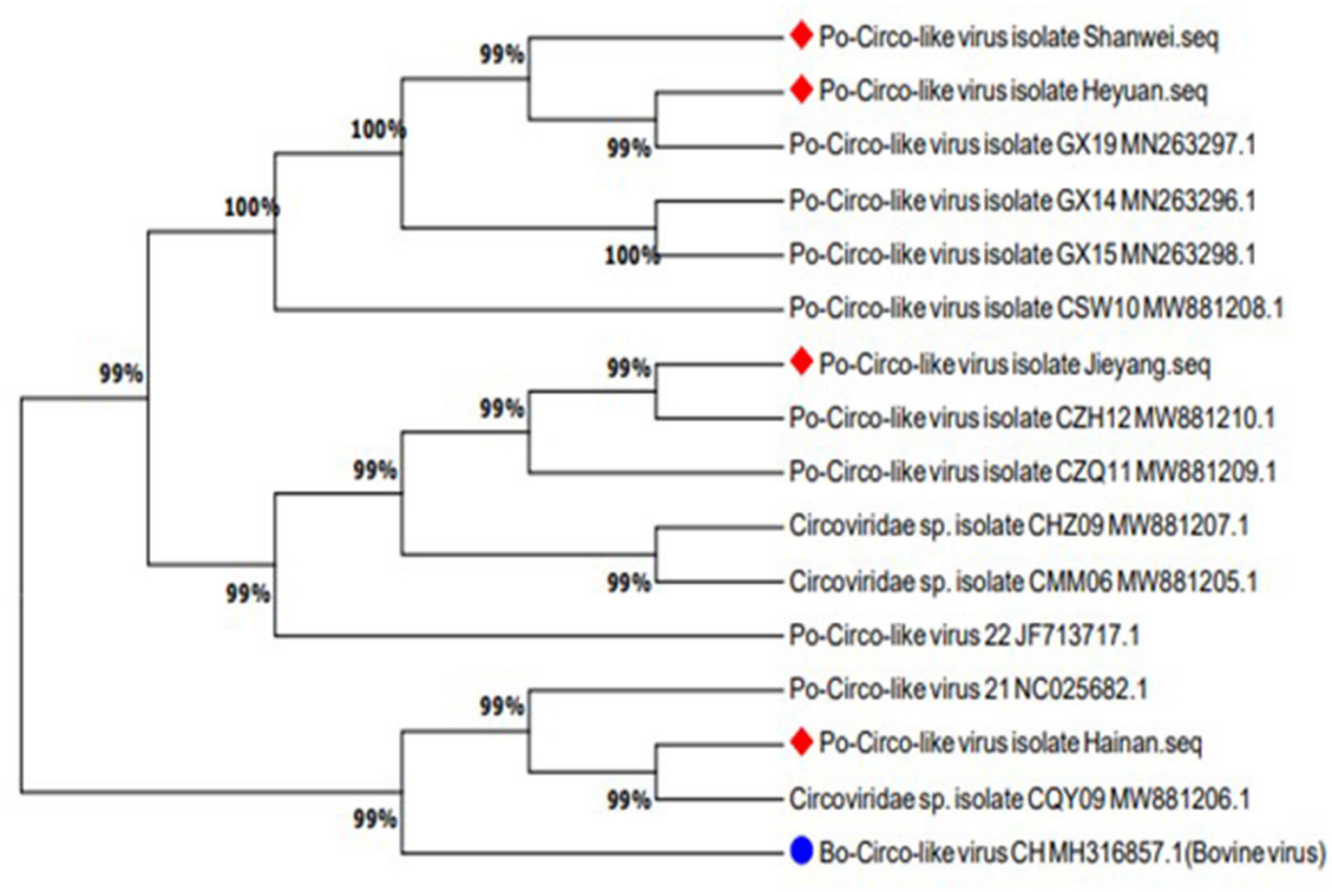
The phylogenetic tree was constructed based on the amino acid sequence of the Rep protein of Porcine Circovirus-like virus from Guangdong, Hainan, and Guangxi provinces of China and the United States. The new strains are marked with circles. Bo-Circo-like virus, identified from a calf with severe hemorrhagic enteritis in China, is marked with a quadrilateral.

**Figure 6:**
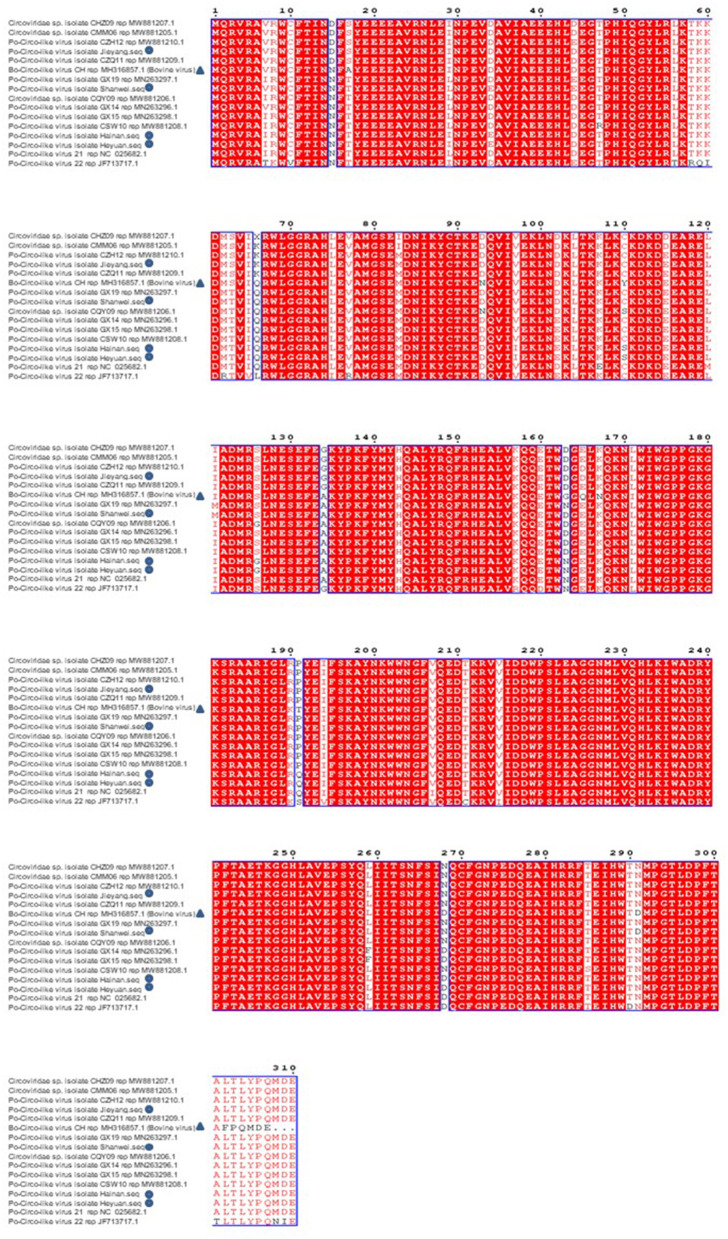
Amino acid comparison and analysis on Rep protein of Porcine Circovirus-like virus from Guangdong, Hainan, and Guangxi provinces of China and the United States. The new strain is marked by triangles. Bo-Circo-like virus, identified from a calf with severe hemorrhagic enteritis in China, is also included.

Sequences uploaded to GenBank from Guangdong, Hainan, and Guangxi provinces of China and the United States were used to construct a phylogenetic tree based on the nucleotide sequence of PCL ORF1 (Figure 5). Previous studies have shown that PCL virus strains we detected had 89% homology with Bo-Circo-like virus from Bovine. In this study, four PCL virus strains showed a sequence similarity of 86.7%–87.8% for the rep gene and 92.2%–92.9% for the Rep protein with Bo-Circo-like virus (Figure 6), demonstrating further the two viruses from swine and bovine share high homology.

## Discussion

**Figure 7:**
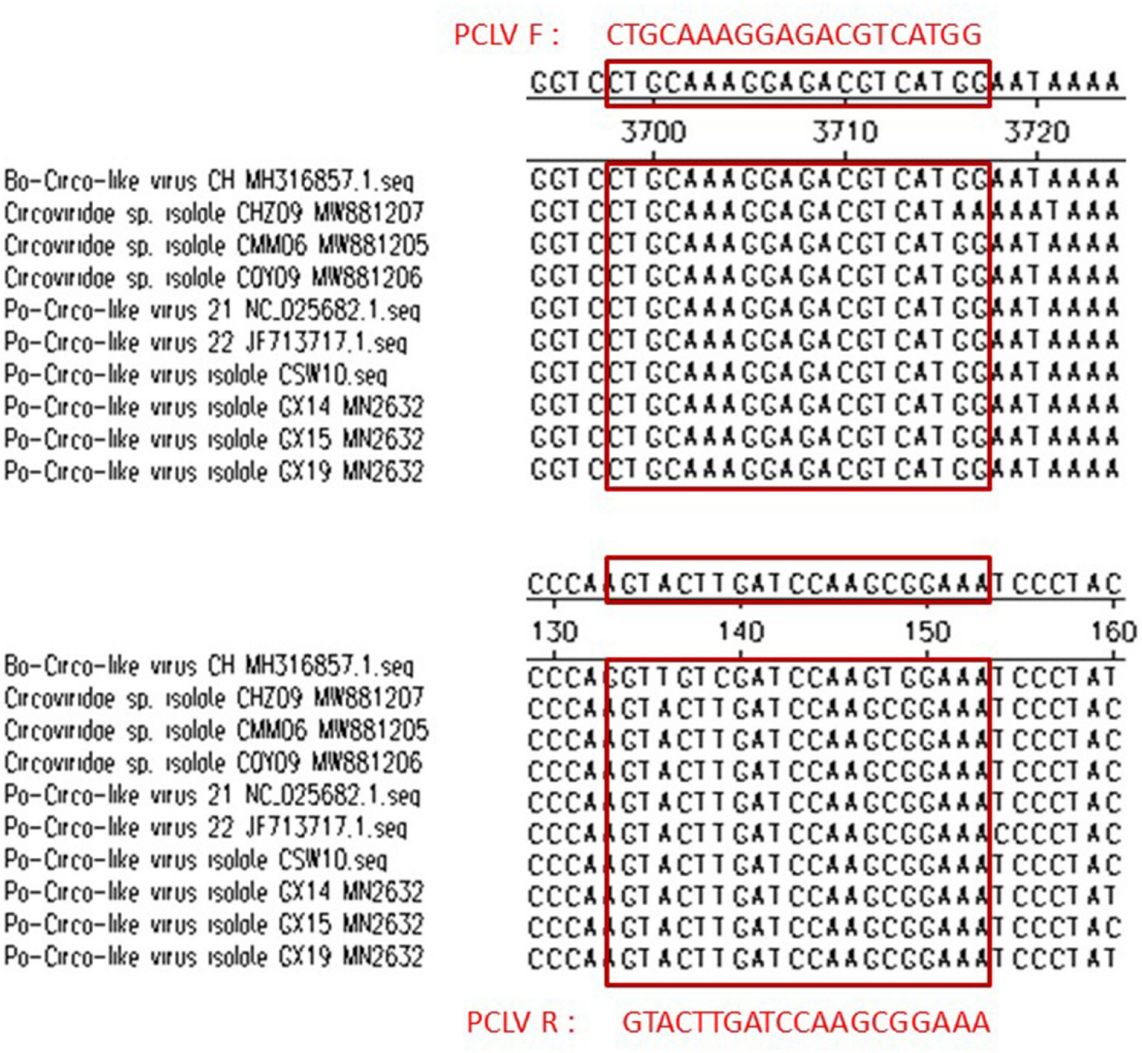
The location of the primers and their comparison with the published PCLV gene sequences.

In this study, in the concentration range of 1.7 × 10^8^ copies/μl−1.7 × 10^2^ copies/μl, a standard curve with a coefficient of determination of 0.999 was plotted with the standard deviations of cycle threshold value in both intra- and inter-assays, suggesting that the reproducibility of the experiment is within an acceptable range. Phylogenetic tree analysis and sequence alignment were carried out based on the sequence ORF1 sequence of the virus strain found in China and the United States, and found that each strain has good homology but also shows a few mutated genes, which will be a challenge for primer design. To satisfy the specificity and avoid the risk of the off-target due to the differences in base sequences of various strains, primers were successfully designed by comparing the genome sequences of published strains (Figure 7). The primers were used to test the positive samples of Guangdong, Jiangxi, and Hainan Provinces stored in our laboratory, and both showed a good amplification curve, verifying its feasibility. We also detected positive cases using our established method in a pig farm in Jiangxi Province, but the full gene sequence was not amplified due to low viral titers. In conclusion, the specificity, sensitivity, application, and reproducibility of a SYBR green-based real-time quantitative PCR test for PCL virus detection were examined in this work. In clinical application, from June to August 2021, 138 fecal samples were collected from diarrheal pig farms in South China. Approximately, 22.46% (31/138) of the samples and 40% (8/20) of the pig farms were positive for PCL virus. Furthermore, we detected positive cases for the first time in Hainan and Jiangxi provinces. This means that the virus may be spreading and existing widely in southern China.

Previous studies have found that the virus can be detected in pig blood, lungs, intestines, lymph nodes, and feces. However, viral titers were highest in the watery feces and jejunum, ileum, and rectum tissues, so priority samples for testing are feces and the posterior intestinal tract tissues. Recently, with further epidemiological investigation, positive nucleic acid has been detected in the umbilical cord blood of aborted stillborn fetus as well (Table 3), which indicates that the disease may have the same possibility of vertical transmission as Porcine Epidemic Diarrhea Virus (PEDV), and may even cause reproductive disorders (19). Of course, due to the possibility of fecal contamination in the process of sample collection, the result still needs further verification.

**Figure 8:**
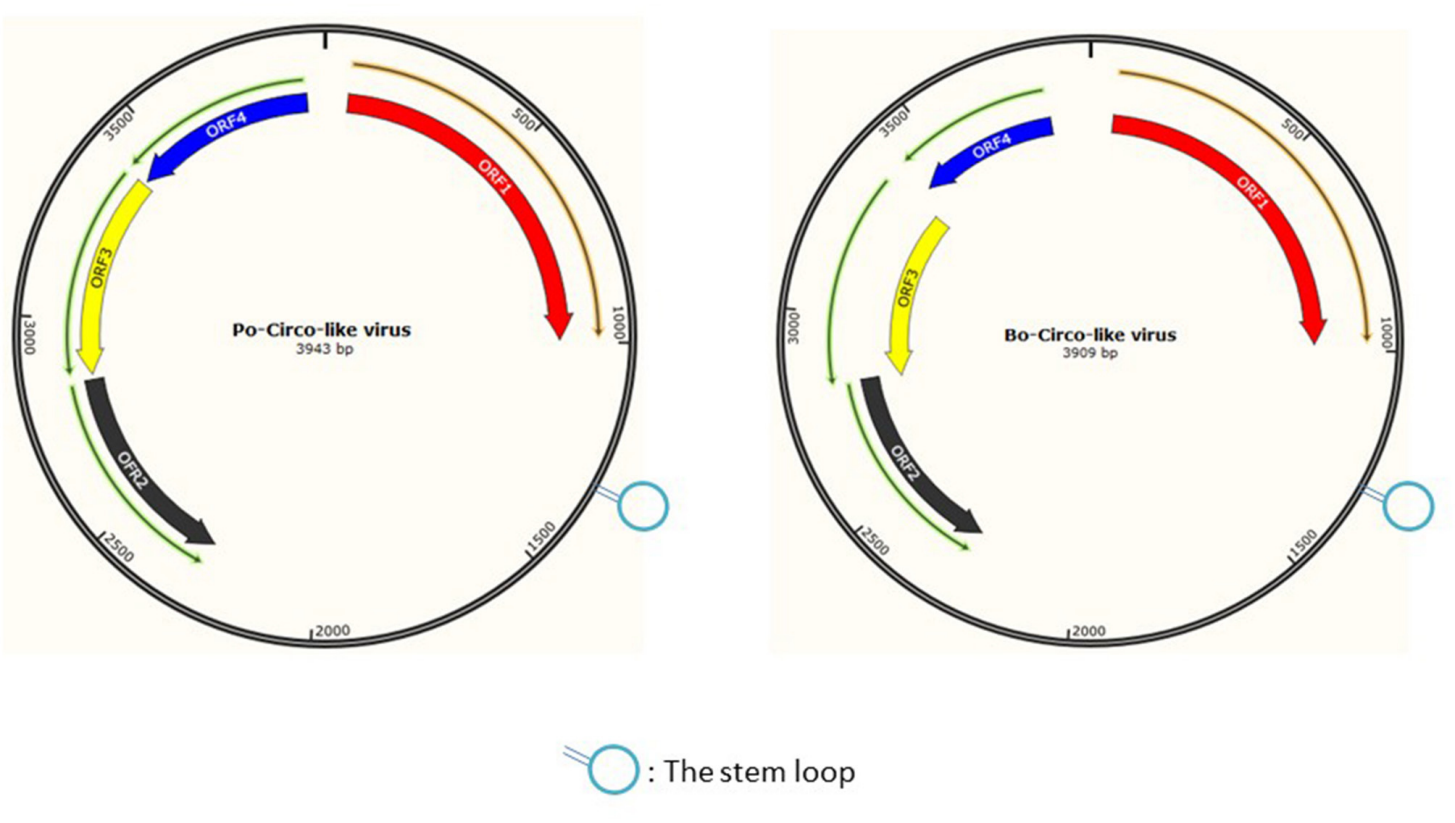
Comparison of genome structure of Po-Circo-like virus and Bo-Circo-like virus.

Porcine circovirus-like virus is a single-stranded DNA virus with a genome of approximately 3.9 kb, which is initiated and encoded by the Rep proteins, and the rolling-circle mechanism is used to replicate (20). The PCL virus's stem loop has one nucleotide substitution, which would create the conditions for its genetic recombination or mutation (8). Interestingly, our laboratory found that the six PCL virus strains from pigs had a sequence similarity of 86.7%–87.8% for the rep gene and sequence similarity of 92.2%–92.9% for the Rep protein with that of Bo-Circo-like virus that is bovine origin. Furthermore, PCLV and Bo-Circo-like virus had similar stem-loop sequences (Figure 8), and this structure is closely associated with virus mutation and cross-species transmission (16). So it is difficult to guarantee that the virus will not become more mutated and spread across species. And piglet diarrhea has been the most important problem plaguing the pig industry, by which the PCLV could be involved. Therefore, there is still a long way to go to understand the origin, isolation, identification, and pathogenicity of PCLV.

In this study, we offer an effective diagnostic tool for PCL virus detection and epidemiological investigation, and provides evidence for the mutation and epidemic of the virus in South China as well, and warns the appropriate measures for prevention and control of the infection of this virus should be under consideration in the near future.

## Data Availability Statement

The datasets presented in this study can be found in online repositories. The names of the repository/repositories and accession number(s) can be found in the article/supplementary material.

## Author Contributions

LZ was responsible for collecting experimental samples, sorting out data, and writing the article. XZ was responsible for experimental design and revising the article. CS provided financial support and revising the article. All authors contributed to the article and approved the submitted version.

## Funding

This work was funded by development of a gene deletion vaccine for African swine Fever (2021YFD1801205).

## Conflict of Interest

ZL was employed by Dongrui Food Group Co. Ltd. GH was employed by Daguang Food Group Co. Ltd. The remaining authors declare that the research was conducted in the absence of any commercial or financial relationships that could be construed as a potential conflict of interest. The reviewer S-ZL declared a shared affiliation with the authors to the handling editor at the time of review.

## Publisher's Note

All claims expressed in this article are solely those of the authors and do not necessarily represent those of their affiliated organizations, or those of the publisher, the editors and the reviewers. Any product that may be evaluated in this article, or claim that may be made by its manufacturer, is not guaranteed or endorsed by the publisher.
